# Supplementary material for: The Construction and Exploration of a Comprehensive MicroRNA Centered Regulatory Network in Foxtail Millet (Setaria italica L.)
Source: Front Plant Sci. 2022 May 6;13:848474. doi: 10.3389/fpls.2022.848474 (PMC9121102; doi:10.3389/fpls.2022.848474)
Supplement: Supplementary file 2 [file Data_Sheet_2.zip › Sit-miR827_Seita.3G030600.1_1467_TPlot.pdf]

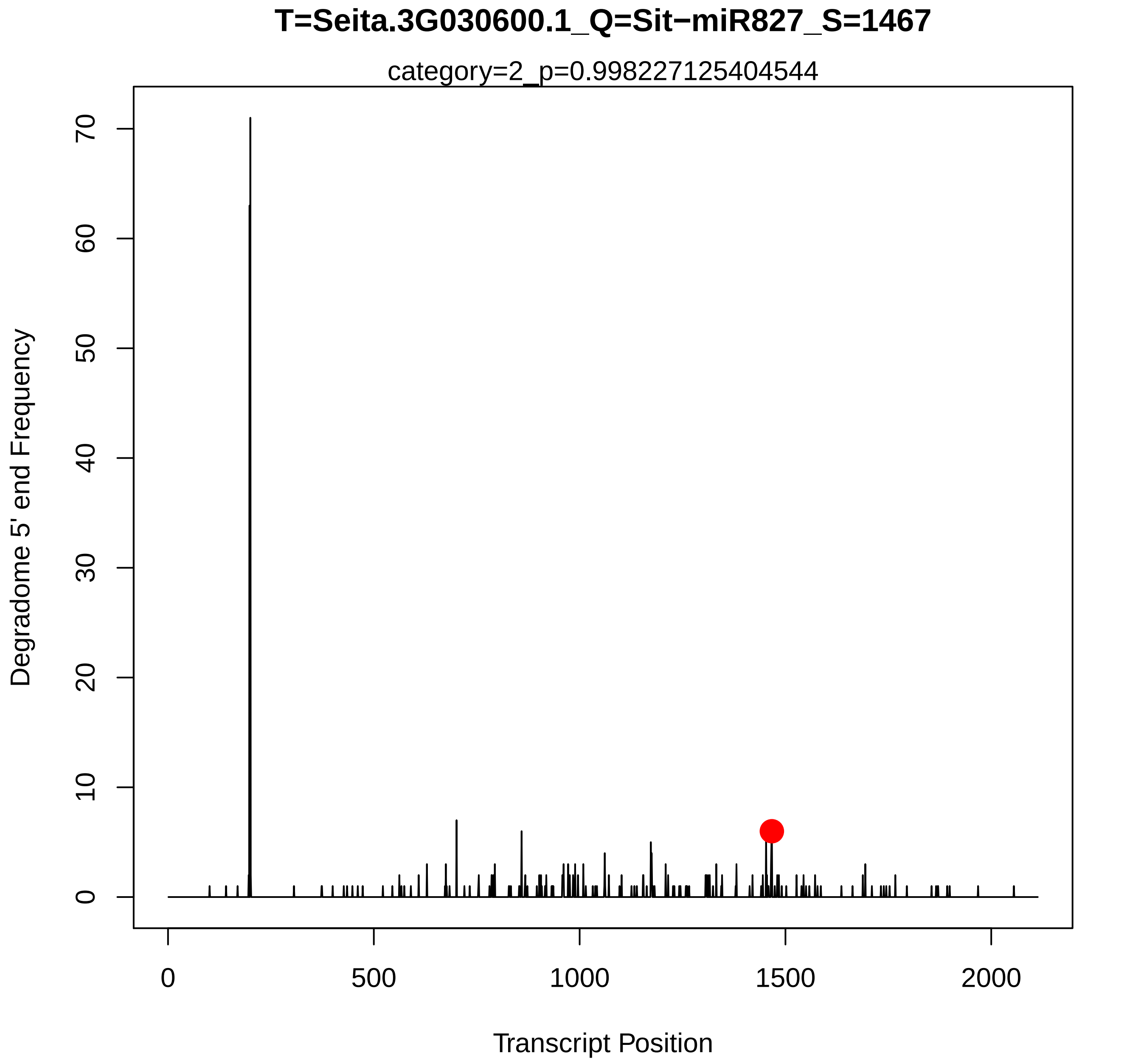
T=Seita.3G030600.1_Q=Sit−miR827_S=1467
category=2_p=0.998227125404544
Degradome 5' end Frequency
0
10
20
30
40
50
60
70
Transcript Position
0
500
1000
1500
2000
